# Physical Activity Might Reduce the Adverse Impacts of the FTO Gene Variant rs3751812 on the Body Mass Index of Adults in Taiwan

**DOI:** 10.3390/genes10050354

**Published:** 2019-05-09

**Authors:** Yi-Ching Liaw, Yung-Po Liaw, Tsuo-Hung Lan

**Affiliations:** 1 School of Nutrition and Health Sciences, Taipei Medical University, Taipei 11031, Taiwan; b506100085@tmu.edu.tw; 2 Institute of Clinical Medicine, National Yang-Ming University, Taipei 11221, Taiwan; 3 Department of Public Health and Institute of Public Health, Chung Shan Medical University, No. 110, Sec. 1 Jianguo N. Road, Taichung 40201, Taiwan; 4 Department of Family and Community Medicine, Chung Shan Medical University Hospital, Taichung 40201, Taiwan; 5 Department of Psychiatry, Taichung Veterans General Hospital, 1650 Taiwan Boulevard Sect. 4, Xitun District, Taichung 407, Taiwan

**Keywords:** body mass index, Taiwan biobank, obesity, physical exercise

## Abstract

The fat mass and obesity-associated (*FTO*) gene is a significant genetic contributor to polygenic obesity. We investigated whether physical activity (PA) modulates the effect of *FTO* rs3751812 on body mass index (BMI) among Taiwanese adults. Analytic samples included 10,853 Taiwan biobank participants. Association of the single-nucleotide polymorphism (SNP) with BMI was assessed using linear regression models. Physical activity was defined as any kind of exercise lasting 30 min each session, at least three times a week. Participants with heterozygous (TG) and homozygous (TT) genotypes had higher BMI compared to those with wild-type (GG) genotypes. The β value was 0.381(*p* < 0.0001) for TG individuals and 0.684 (*p* = 0.0204) for TT individuals. There was a significant dose-response effect among carriers of different risk alleles (*p* trend <0.0001). Active individuals had lower BMI than their inactive counterparts (β = −0.389, *p* < 0.0001). Among the active individuals, significant associations were found only with the TG genotype (β = 0.360, *p* = 0.0032). Inactive individuals with TG and TT genotypes had increased levels of BMI compared to those with GG genotypes: Their β values were 0.381 (*p* = 0.0021) and 0.950 (*p* = 0.0188), respectively. There was an interaction between the three genotypes, physical inactivity, and BMI (*p* trend  = 0.0002). Our data indicated that increased BMI owing to genetic susceptibility by *FTO* rs3751812 may be reduced by physical activity.

## 1. Introduction

Obesity is a global public health issue associated with an unhealthy lifestyle and genetic factors. According to the World Health Organization (WHO), more than 1.9 billion adults are either overweight or obese [1]. Based on criteria established by the Department of Health in Taiwan, a body mass index (BMI) of 24–26.9 kg/m^2^ indicates overweight while BMI ≥ 27 kg/m^2^ indicates obesity. Results from the 1993–1996 and 2005–2008 Nutrition and Health Surveys showed an increased prevalence of obesity and overweight among Taiwanese individuals (that is, 33.4–51% in men and 33.5–35.9% in women) [2]. About 17.1% of children and adolescents in the United States were overweight, while 32.2% of adults were obese in 2003–2004 [3]. The etiology of obesity is complex and multifactorial, involving genetic background, hormones, and lifestyle and environmental issues [4].

The fat mass and obesity-associated (*FTO*) gene variants rs1121980, rs17817449, rs8050136, rs9935401, rs3751812, rs9939609, rs9930506, and rs9922708 were previously associated with obesity [5,6]. Several other variants have been associated with obesity risk in different population and age groups [7,8,9,10,11]. The role played by *FTO* and other obesity-related genes in the etiology of obesity has been reported [12]. The mechanisms responsible for the effect of the *FTO* gene on obesity remain unknown [13]. Multiple single-nucleotide polymorphisms (SNPs) in the first intron of the *FTO* gene have been associated with BMI [14]. Because the intron lacks protein-altering variants and the biological pathways involved are not well-known, several studies have provided a candidate mechanism of obesity and *FTO* [15,16,17,18,19].

Physical activity (PA) prevents obesity in many ways. Several studies have associated the *FTO* gene with PA. In one of its variants, rs1477196, the C allele has been associated with a 1.22 increase in BMI among Old Order Amish (OOA) individuals who were engaged in low levels of physical activity [20]. However, only a 0.27 increase was noted in people who were engaged in high levels of physical activity. In the same study, the difference in BMI across rs1861868 genotypes was large in less physically active individuals but was small (although not significant) in the more physically active individuals. In another study, A-allele homozygotes who were physically inactive had a 1.95 increase in BMI compared with T-allele homozygotes [21].

The European Prospective Investigation into Cancer and Nutrition-Norfolk Study also confirmed that physical activity attenuated the effect of rs1121980 on BMI. However, in the physically active group, the risk allele increased BMI by 0.25 per allele. The increase was significantly more pronounced in inactive individuals (0.44 per risk allele) [22]. However, other studies found no interaction between BMI and physical activity [23,24,25].

Based on previous studies, *FTO* gene effects on obesity may be modified by physical activity [26,27]. In Taiwan, obesity (defined by a BMI at or greater than 27 kg/m^2^) is a serious health issue. How physical activity modulates the BMI-increasing influence of *FTO* variants in Asian populations is not well-understood. Therefore, the purpose of this study was to examine the role PA plays in modifying the effect of *FTO* variants on BMI. We hypothesized that increased BMI due to genetic susceptibility by an *FTO* variant (rs3751812) may be attenuated by physical activity.

## 2. Materials and Methods

### 2.1. Participants and Measurements

Study data were obtained from Taiwan biobank, a national large-scale data source with genetic and demographic data from Taiwanese individuals between the ages of 30 and 70. Initially, data were collected from 10,853 participants. After excluding those with incomplete information (n = 21), the final enrollment included 10,832 participants. Anthropometric measures included body weight and height (measured in accordance with the standard procedures), as well as BMI (weight/height^2^). Other variables included age, sex, physical activity/inactivity, total cholesterol, smoking (defined as never/former and current smoking), alcohol consumption (defined as weekly drinking of at least 150 cc of alcohol continuously for 6 months), vegetarian diet (defined as never/former and current vegetarian), coffee intake (that is, more than three times per week), and tea consumption (more than one time per day). Physical activity was defined as any kind of exercise lasting 30 min each session, at least three times a week. All study participants provided written informed consent, according to protocols approved by the institutional review board. All methods were carried out in accordance with relevant guidelines and regulations. The Institutional Review Board of Chung Shan Medical University approved this study (CS2-16114, 18 October 2016).

### 2.2. Genotyping

Eight SNPs in the *FTO* gene that have been consistently associated with obesity in European populations were selected. The source of SNPs was the human genome database (accessed at https://www.ncbi.nlm.nih.gov/genome/guide/human/), which contains findings from international research programs, such as the HapMap and 1000 Genomes Projects. Included in our analysis were the following variants: rs1121980, rs17817449, rs8050136, rs9935401, rs3751812, rs9939609, rs9930506, and rs9922708. SNPs were excluded if their minor allele frequencies (MAFs) were less than 0.01, or call rates less than 98%. Also excluded were SNPs that were not in Hardy–Weinberg equilibrium. In the final model, one tagging SNP (rs3751812) was selected for genotyping.

### 2.3. Statistical Analysis

All analyses were conducted using SAS 9.3 statistical software (SAS Institute, Cary, NC, USA). The Χ^2^ test was used to compare the differences between the three genotypes. Data were expressed as X ± standard error (S.E.) and %. Multicollinearity was measured using the variance inflation factor (VIF). Values that exceeded 10 suggested multicollinearity. Hardy–Weinberg equilibrium (HWE) was tested for each SNP using a 1 degree of freedom χ^2^-test. LD and its correlation coefficients (D values) were calculated using the Haploview software. Linear regression models were used to test the association between the tag-SNP and BMI. Adjustments were made for potential confounding variables (age, sex, physical activity, alcohol drinking, smoking, total cholesterol, tea consumption, coffee consumption, and vegetarian diets). *p*  <  0.05 was considered statistically significant.

## 3. Results

Demographic characteristics of participants are shown in Table 1. Among individuals with the *FTO* rs3751812 variant, 168 (1.55%) were homozygous (TT), 2343 (21.63%) were heterozygous (TG), and 8321 (76.82%) were wild-type (GG). The mean BMI was 25.21 ± 0.26 kg/m^2^ for TT carriers, 24.58 ± 0.08 kg/m^2^ for TG carriers, and 24.27 ± 0.04 kg/m^2^ for GG carriers. There were more women with GG and TG genotypes than men (that is 51.91% versus 48.06% for GG and 52.80% versus 47.20% for TG genotype) except for those with the TT genotype (that is 39.88% versus 60.12%). Fasting blood glucose levels differed significantly across genotypes (*p* = 0.0398). However, values were within normal ranges. Other variables did not differ significantly across genotypes. Table 2 shows the demographic and lifestyle variables of study participants based on physical exercise. Among physically active individuals, 66 (1.49%) were homozygous (TT), 975 (22.07%) were heterozygous (TG), and 3376 (76.43%) were wild-type (GG). Likewise, among their inactive counterparts, 102 (1.59%) were homozygous (TT), 1368 (21.33%) were heterozygous (TG), and 4944 (77.08%) were wild-type (GG). There were no significant differences between physically active and inactive participants based on genotype distributions (*p* = 0.6154). Fasting glucose and total cholesterol differed among physically active and inactive individuals though values were within normal ranges. Table 3 shows the association between rs3751812 and BMI. After adjusting for potential confounders, individuals with TG and TT genotypes exhibited higher BMI compared to those with GG genotypes. Their β values were β = 0.381 (*p* < 0.0001) and 0.684 (*p* = 0.0204), respectively. BMI significantly increased with an increase in the number of risk alleles (*p* trend <0.0001). In addition, active individuals had a lower BMI than their inactive counterparts (β = −0.389, *p* < 0.0001). Table 4 shows the association of analyzed variables with BMI in groups with different genotypes. Decreased BMI was associated with GG (β = −0.368, *p* < 0.0001) and TG (β = −0.414, *p* = 0.0175) carriers who were physically active compared to their physically inactive counterparts. However, the decreased BMI in TT carriers was not significant (β = −1.059, *p* = 0.1099). This may have been due to the small sample size.

Table 5 shows the association between rs3751812 and BMI based on exercise status. Among physically active individuals, the TG genotype was significantly associated with increased BMI (β = 0.360, *p* = 0.0032). However, the effect of the TT genotype on BMI was not significant (β = 0.245, *p* = 0.5606). Among physically inactive individuals, both TG (β = 0.381, *p* = 0.0021) and TT (β = 0.95036, *p* = 0.0188) were significantly associated with increased BMI (*p* trend = 0.0002). However, no dose-related trend was observed among physically active individuals.

## 4. Discussion

In the current study, we found that there was an association between *FTO* SNP (rs3751812) and BMI among Taiwanese adults. Compared with the GG genotype, carriers of the TT genotype had a higher BMI than those with the GT genotype. Similar findings were reported among the Han Chinese, where increased mean values of BMI were seen among GT + TT than GG carriers [28]. In addition, we found that the effect on BMI was in an allele-dose-dependent manner (*p* trend < 0.0001).

After stratification, we found that physical activity was significantly associated with a decreased BMI. The decrease was significant in carriers of GG and TG genotypes. Carriers of the TT genotype also exhibited decreased BMI even though the effect was not significant. Latinos with TT alleles (considered as carriers of two risk alleles) who were engaged in regular PA exhibited significant reductions in BMI [26].

Efforts have been made to understand the biological mechanisms underlying body weight regulation: SNPs in FTO are believed to be associated with obesity through an effect on RPGRIP1L [29]. Reports from another study indicated that RPGRIP1L may be partly or exclusively responsible for the obesity susceptibility signal at the *FTO* locus [16]. It has also been suggested that the homeobox gene Iroquois homeobox 3 (IRX3) is a functional long-range target of obesity-associated variants within *FTO* [17].

Genetic, environmental, and lifestyle factors affect body mass index. Such variables as tea, coffee, and cholesterol were included in the current study to understand their modulating role on BMI. These variables were selected based on previous associations with obesity. Findings from a previous study showed that the risk of hypercholesterolaemia was modified by BMI in adults aged 25–39 years [30]. Unlike green tea, coffee consumption has been strongly associated with a higher blood cholesterol and BMI [31]. Findings from another study showed that higher coffee drinking attenuated genetic associations with BMI and obesity [32]. Further analysis of our data showed that vegetarian diet was associated with a lower BMI mainly among participants with the GG genotype, as well those that were physically active.

To our knowledge, studies investigating the interactive influence of genes and physical activity have focused on populations other than Asians [26,27,33]. Associations between *FTO* variants with obesity risk (measured by BMI) have not been widely investigated in Taiwan. Therefore, our findings are relevant and may serve as a reference for future studies.

Recent studies have also examined the interactive influence of *FTO* variants and lifestyle factors on obesity risk. Lower levels of BMI have been reported among smokers compared to nonsmokers [34,35]. However, we observed a significant association between smoking and increased BMI (β = 0.501, *p* < 0.0021). This may be associated with other health risks in individuals engaged in heavy smoking. There was no significant association between alcohol intake and BMI. After our stratified analysis, coffee consumers who were engaged in physical activity exhibited increased BMI. Lower levels were observed among consumers that were inactive, though not significant. In addition, we found that vegetarian diet was associated with a decreased BMI. Contrasting results have been reported. According to a study conducted in North America, the mean BMI was lowest in vegans (23.6 kg/m^2^) and was incrementally higher in lacto-ovo vegetarians (25.7 kg/m^2^), pesco-vegetarians (26.3 kg/m^2^), semi-vegetarians (27.3 kg/m^2^), and nonvegetarians (28.8 kg/m^2^) [36]. Our data suggested that BMI is associated with physical activity and *FTO* rs3751812 variants in Taiwanese individuals. The strength of the study included the use of a large-scale data source with genetic and demographic information. In addition, we included information on smoking and drinking habits. However, the study is limited in that we did not consider the gene–gene interaction and the effect on BMI.

In conclusion, our study validated the association between an *FTO* variant and BMI in Taiwanese individuals. In addition, individuals with TG and TT genotypes who were physically active had a decreased BMI. These results indicate that physical activity might be necessary to mitigate the deleterious effect of BMI among genetically susceptible Taiwanese individuals.

## Figures and Tables

**Table 1:** Characteristics of study participants according to rs3751812 genotypes.

Parameters	Total	GG (n = 8321)	TG (n = 2343)	TT (n = 168)	*p*-Value
Age (years)	48.68 ± 0.11	48.64 ± 0.12	48.78 ± 0.23	49.13 ± 0.90	0.7433
BMI (kg/m^2^)	24.35 ± 0.03	24.27 ± 0.04	24.58 ± 0.08	25.21 ± 0.26	<0.0001
Fasting blood glucose(mg/dl)	96.49 ± 0.20	96.36 ± 0.23	96.69 ± 0.43	100.43 ± 2.18	0.0398
Total cholesterol (mg/dl)	193.76 ± 0.34	193.93 ± 0.39	193.10 ± 0.74	194.58 ± 2.43	0.5763
Sex (n, %)					
Male	5219 (48.09)	4002 (48.06)	1106 (47.20)	101 (60.12)	0.0053
Female	5634 (51.91)	4319 (51.90)	1237 (52.80)	67 (39.88)	
Alcohol intake					
Never/Former	9992 (92.08)	7641 (91.84)	2180 (93.04)	151 (89.88)	0.0930
Current	860 (7.92)	679 (8.16)	163 (6.96)	17 (10.12)	
Smoking					
No	7451 (68.70)	5743 (69.50)	1583 (67.65)	111 (66.07)	0.3299
Yes	3395 (31.30)	2574 (30.95)	757 (32.35)	57 (33.93)	
Physical activity					
No	6426 (59.21)	4944 (59.42)	1368 (58.39)	102 (60.71)	0.6154
Yes	4426 (40.79)	3376 (40.58)	975 (41.61)	66 (39.29)	
Tea consumption					
No 5655 (63.06)		4353 (63.26)	1213 (62.82)	78 (56.52)	0.2591
Yes	3312 (36.94)	2528 (36.74)	718 (37.18)	60 (43.48)	
Coffee consumption					
No	6086 (67.87)	4668 (67.84)	1320 (68.36)	84 (60.87)	0.1910
Yes	2881 (32.13)	2213 (32.16)	611 (31.64)	54 (39.13)	
Vegetarian Diet					
Never/Former	8551 (95.36)	6570 (95.48)	1836 (95.08)	129 (93.48)	0.4335
Current	416 (4.64)	311 (4.52)	95 (4.92)	9 (6.52)	

All variables are presented as mean ± standard error (S.E.) (continuous variables) or numbers (%). BMI: body mass index, GG: wild-type, TG: heterozygous; TT: homozygous.

**Table 2:** Demographic and lifestyle variables of study participants under stratification based on physical activity.

Parameters	Physically Active (n = 4426)	Physically Inactive (n = 6426)	*p*-Value
rs3751812 (n, %)			
GG	3376 (76.43)	4944 (77.08)	0.6154
TG	975 (22.07)	1368 (21.33)	
TT	66 (1.49)	102 (1.59)	
Age (years)	53.36 ± 0.15	45.45 ± 0.13	<0.0001
BMI (kg/m^2^)	24.24 ± 0.05	24.42 ± 0.05	0.0083
Fasting glucose (mg/dl)	97.93 ± 0.32	95.50 ± 0.26	<0.0001
Total cholesterol (mg/dl)	195.68 ± 0.53	192.44 ± 0.45	<0.0001
Sex (n, %)			
Male	2158 (48.76)	3061 (47.63)	0.2500
Female	2268 (51.24)	3365 (52.37)	
Alcohol drinking			
Never/Former	4080 (92.18)	5911 (92.00)	0.7294
Current	346 (7.82)	514 (8.00)	
Smoking			
No	3144 (71.05)	4306 (67.07)	<0.0001
Yes	1281 (28.95)	2114 (32.93)	
Tea consumption			
No	2340 (62.37)	3315 (63.57)	0.2455
Yes	1412 (37.63)	1900 (36.43)	
Coffee consumption			
No	2581 (68.79)	3505 (67.21)	0.1140
Yes	1171 (31.21)	1710 (32.79)	
Vegetarian diet			
Never/Former	3598 (95.90)	4953 (94.98)	0.0411
Current	154 (4.10)	262 (5.02)	

**Table 3:** Linear regression analysis showing the association between rs3751812 and BMI.

	β	*p*-Value
rs3751812		
GG		-
TG	0.381	<0.0001
TT	0.684	0.0204
	*p* for trend < 0.0001	
Physical activity	−0.389	<0.0001
Sex	1.384	<0.0001
Age	0.020	<0.0001
Total cholesterol	0.008	<0.0001
Alcohol intake	0.092	0.5267
Smoking	0.501	<0.0001
Tea consumption	0.492	<0.0001
Coffee consumption	0.108	0.1723
Vegetarian diet	−0.343	0.0493

β: beta coefficient.

**Table 4:** Association of analyzed variables with BMI in groups with different genotypes.

	GG		TG		TT	
	β	*p*-Value	β	*p*-Value	β	*p*-Value
Physical activity	−0.368	<0.0001	−0.414	0.0175	−1.059	0.1099
Sex	1.461	<0.0001	1.196	<0.0001	0.850	0.1984
Age	0.019	<0.0001	0.026	0.0013	0.014	0.6146
Total cholesterol	0.008	<0.0001	0.008	0.0003	0.004	0.6724
Alcohol intake	0.060	0.7148	−0.008	0.9819	2.065	0.0337
Smoking	0.387	0.0003	0.860	<0.0001	0.885	0.2209
Tea consumption	0.488	<0.0001	0.516	0.0031	0.499	0.3737
Coffee consumption	0.212	0.0179	−0.215	0.2260	−0.641	0.2823
Vegetarian diet	−0.516	0.0098	0.166	0.6626	0.401	0.7291

**Table 5:** Association between rs3751812 and obesity based on physical activity.

	Physical Activity		Physical Inactivity	
	β	*p*-Value	β-	*p*-Value
rs3751812				
GG	-	-	-	-
TG	0.360	0.0032	0.381	0.0021
TT	0.245	0.5606	0.950	0.0188
				*p* trend = 0.0002
Sex	1.150	<0.0001	1.517	<0.0001
Age	0.006	0.2077	0.030	<0.0001
Total cholesterol	0.003	0.0741	0.011	<0.0001
Alcohol drinking	0.460	0.0241	−0.192	0.3374
Smoking	0.439	0.0012	0.514	<0.0001
Tea consumption	0.565	<0.0001	0.417	0.0001
Coffee consumption	0.270	0.0150	−0.017	0.8746
Vegetarian diet	−0.626	0.0152	−0.224	0.3401

β: beta coefficient.
